# Hunt for the Shunt: An Unusual Case of Late-onset Hydrothorax in a Patient on Peritoneal Dialysis

**DOI:** 10.7759/cureus.3839

**Published:** 2019-01-07

**Authors:** Karan N Ramakrishna, Dhruv Lowe, Uma K Murthy

**Affiliations:** 1 Internal Medicine, State University of New York Upstate Medical University, Syracuse, USA; 2 Gastroenterology, State University of New York Upstate Medical University, Syracuse, USA; 3 Gastroenterology, Syracuse Veteran's Affairs Medical Center, Syracuse, USA

**Keywords:** peritoneal dialysis, hydrothorax, pleuroperitoneal shunt

## Abstract

Hydrothorax is a rare complication of peritoneal dialysis occurring in about 2% of continuous ambulatory peritoneal dialysis (CAPD) patients. These effusions occur soon after the onset of dialysis and are usually right-sided. We describe an unusual case of late-onset, left-sided, and recurrent effusions in the setting of CAPD.

A 67-year-old patient with end-stage renal disease on CAPD for the last three years was admitted to our hospital with acute hypoxic respiratory failure secondary to a left-sided effusion. Although previously asymptomatic, he had three admissions for bilateral (left predominant) effusions in the last year, all of which were found to be transudative on analysis. Therapeutic thoracentesis once again revealed a transudative effusion with an elevated pleural fluid-serum glucose gradient. On this occasion, pleuro-peritoneal scintigraphy with technetium-99m was performed, uncovering a communication between the peritoneal cavity and the left pleural cavity. The peritoneal dialysis was substituted with hemodialysis, and the patient showed an eventual resolution of left-sided effusions within 18 months.

Hydrothorax in peritoneal dialysis is due to the transudation of fluid across congenital or acquired pleuro-peritoneal communications. Pleural fluid with protein content less than 3 g/dl, high glucose, and low lactate dehydrogenase (LDH) relative to blood, and the presence of both D and L isomers of lactic acid suggest a transdiaphragmatic leak. Early diagnosis via peritoneal scintigraphy and appropriate management can prevent significant morbidity and mortality.

## Introduction

Hydrothorax is the accumulation of excessive serous fluid in the pleural cavity. It is a rare, but acknowledged, side effect of peritoneal dialysis, having been first described in 1967 [1]. There is a reported occurrence in approximately 2% of continuous ambulatory peritoneal dialysis (CAPD) patients. There have been multiple postulated mechanisms as to the etiology of this complication. The most accepted theory is the transudation of fluid under increased intra-abdominal pressure across previously present congenital anatomic defects in the diaphragm. This also explains the preponderance of right-sided hydrothorax, as it is believed that defects on the left are covered by the heart and pericardium. These leaks can be classified into early (within 30 days) and late (after 30 days) based on the onset of symptoms following the initiation of peritoneal dialysis. Right-sided effusions, occurring soon after the commencement of CAPD, with significantly elevated glucose content, accompanied by unexplained low drainage output from dialysis exchange are all clinical indicators that point towards a diagnosis of hydrothorax associated with a pleuro-peritoneal shunt. Here, we describe an unusual presentation of the above with late-onset and recurrent left-sided effusions.

## Case presentation

A 67-year-old patient with multiple medical comorbidities (diabetes mellitus, hypertension) was admitted for dyspnea and hypoxia. A chest X-ray revealed the cause to be a left pleural effusion. The patient had been diagnosed with end-stage renal disease (ESRD) about three years before and had been on CAPD for approximately 30 months. He had received about six months of hemodialysis via a permacath prior to being transitioned to peritoneal dialysis. Although he responded well to peritoneal dialysis initially, he had three admissions in the past year alone for a similar presentation with bilateral pleural effusions noted on imaging. Thoracentesis on these occasions had indicated transudative fluid with cytology negative for malignancy. A relatively high level of glucose was noted in pleural fluid (140-200 mg/dl). No definite cause was identified, and the patient was discharged after the improvement of symptoms with thoracentesis.

**Figure 1:**
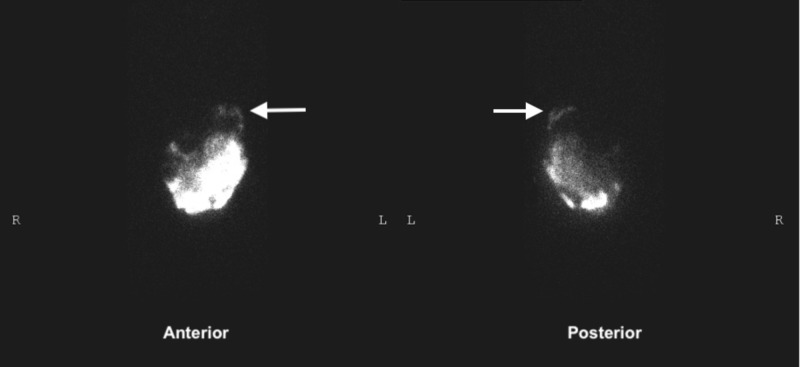
Pleuro-peritoneal scintigraphy demonstrating the trans-diaphragmatic leakage of the dialysate (containing radioactive Tc-99m tracer) and accumulation, predominantly in the left pleural cavity

During his most recent admission, he was in marked respiratory distress, with decreased breath sounds and dullness to percussion in left lower lung base. Pulse oximetry and arterial blood gas sampling indicated hypoxia. A chest X-ray revealed the presence of a moderate left-sided pleural effusion. Thoracentesis was performed and 1.5 L of serous fluid was drained. Fluid analysis revealed the following: pH 7.449, glucose 183 mg/dL, protein 2.5 g/dL, and lactate dehydrogenase (LDH) 71 U/l. Echocardiogram ruled out decompensated congestive heart failure. These recurrent effusions, with no evident cause, were concerning and provoked a suspicion for a possible pleuro-peritoneal shunt in the setting of chronic CAPD. This suspicion was further bolstered when looking at the Chow glucose gradient, which is the difference between glucose levels in pleural fluid and serum. This was found to be 103 mg/dl, strongly indicating a peritoneal origin for the pleural effusion. For confirmation, pleura-peritoneal scintigraphy with technetium sulfur colloid (Tc-99m) was done, which showed a definite communication between the peritoneal cavity and left pleural space (Figure 1).

The patient’s peritoneal dialysis catheter was removed and he was transitioned to hemodialysis with the placement of a permacath. He was continued on outpatient hemodialysis at discharge, which he appeared to tolerate well and continued close outpatient follow-up with nephrology. The patient did not have a similar presentation again. A chest X-ray 18 months after the stoppage of peritoneal dialysis showed the complete absence of any effusion and the presence of only residual scarring and atelectasis.

## Discussion

Numerous clinical, financial, logistic, and socio-environmental factors play a role in deciding on the type of renal replacement therapy. At this time, there is no evidence that shows the relative benefit of one mode of dialysis over the other [2]. Peritoneal dialysis is often chosen as an initial therapy over hemodialysis, as it has the advantages of a better overall quality of life, better control of anemia and bone turnover, and better outcomes in pre-renal transplant patients [3]. Peritoneal dialysis is associated with its own set of complications that can be infectious (common) or non-infectious (less common). The non-infectious complications are usually attributed to the increased intra-abdominal pressure exerted by the large volume of dialysate within the peritoneum.

Hydrothorax in peritoneal dialysis is thought to be due to the movement of fluid across congenital or acquired communications between the pleura and peritoneum under this increased intra-abdominal pressure. The reported incidence varies between 1.6 and 10 percent [4-5]. In a review of 50 patients, Abraham et al. [5] found that the effusions were more common in females, with a median onset of 25 days following the initiation of dialysis and were predominantly right-sided (66%). However, another larger study in Japan (Nomoto et al. [4] ) had demonstrated equal sex predilection and onset of symptoms anywhere between one day and eight years. Again, the predominance of right-sided effusions (88%) was seen. The most common symptoms were dyspnea, weight gain, and diminished ultrafiltration. Protein content of less than 3 g/dl, significantly high pleural fluid sugar, and relatively low LDH in comparison to blood levels, all suggest a transdiaphragmatic leak as the cause of hydrothorax [6-7]. The presence of both D and L isomers of lactic acid (only L isomer is present endogenously in the body) also indicates that the pleural fluid likely contains dialysate. Calculation of the pleural fluid-serum glucose gradient (popularly called the “Chow gradient”) with a level above 100 mg/dl has demonstrated high sensitivity and specificity for the diagnosis of pleuro-peritoneal communication in patients on peritoneal dialysis [7]. Currently, the safest, most effective, and rapid method of diagnosing a peritoneal leak is via peritoneal scintigraphy with technetium isotope. It has the added benefit of determining its location in case shunt surgery is needed. Once a pleuro-peritoneal leak is confirmed, conservative management with the temporary cessation of peritoneal dialysis is effective in about 50% of the cases. CAPD can be reinstated with a modified regimen (intermittent or with a smaller volume and shorter duration) after a resolution of hydrothorax. In patients who do not respond to the above, a surgical approach with chemical pleurodesis or diaphragmatic repair (open or video-assisted thoracotomy) is indicated [8].

## Conclusions

The case that we have described illustrates a rare presentation of pleural effusion due to a peritoneal leak. In this instance, the onset of symptoms was more than two years after the initiation of CAPD and the recurrent effusions were unusually left-sided. This may have played a role in decreasing the suspicion of clinicians for suspecting a pleuro-peritoneal shunt. Early pleuro-peritoneal scintigraphy and diagnosis of this communication followed by appropriate management (including stoppage of peritoneal dialysis, pleurodesis, or surgical correction) may have avoided multiple hospitalizations and significant morbidity. With the increasing use of peritoneal dialysis across the United States and Europe, it is important to recognize the varied clinical presentation of the pleuro-peritoneal shunting of the dialysate in order to facilitate early diagnosis and decrease the significant burden on morbidity and mortality.
